# The genome sequence of the Australian filarial nematode, *Cercopithifilaria johnstoni*

**DOI:** 10.12688/wellcomeopenres.17258.1

**Published:** 2021-10-12

**Authors:** Kirsty McCann, Warwick Grant, Stephen R. Doyle

**Affiliations:** 1 Department of Physiology, Anatomy & Microbiology, La Trobe University, Bundoora, Australia; 2 Parasites & Microbes Programme, Wellcome Sanger Institute, Hinxton, Cambridgeshire, CB10 1SA, UK

**Keywords:** Cercopithifilaria johnstoni, filarial nematode, genome assembly, Illumina MiSeq

## Abstract

We present a genome assembly and annotation of an individual female *Cercopithifilaria johnstoni*, a parasitic filarial nematode that is transmitted by hard ticks (Ixodidae) to infect a broad range of native Australian murid and marsupial hosts. The genome sequence is 76.9 Mbp in length, and although in draft form (N50 = 99 kbp, N50[n] = 232), is largely complete based on universally conserved orthologs (BUSCOs; genome = 94.9%, protein = 96.5%) and relative to other related filarial species. These data represent the first genomic resources for the genus *Cercopithifilaria*, a group of parasites with a broad host range, and form the basis for comparative analysis with the human-infective parasite, *Onchocerca volvulus*, both of which are responsible for similar eye and skin pathologies in their respective hosts.

## Species taxonomy

Eukaryota; Opisthokonta; Metazoa; Eumetazoa; Bilateria; Protostomia; Ecdysozoa; Nematoda; Chromadorea; Rhabditida; Spirurina; Spiruromorpha; Filarioidea; Onchocercidae, Cercopithifilaria, *Cercopithifilaria johnstoni* (taxon ID: 2874296)

## Introduction

*Cercopithifilaria johnstoni* ( Mackerras, 1954) is a parasitic filarial nematode transmitted by ixodid ticks to infect a diverse range of native Australian mammalian hosts ( Spratt & Haycock, 1988), including monotremes, marsupials, and native rodents. The ability to infect such a broad host range is unusual for a filarial parasite; however, it is yet to be determined if this reflects permissive infectivity and persistence in diverse hosts or cryptic species diversity among morphologically indistinguishable parasites. Over 30 years ago, investigation of *C. johnstoni* infection of native hosts and experimentally-infected laboratory rats ( *Rattus norvegicus*) revealed that *C. johnstoni* could cause skin and ocular immunopathologies that appear to be analogous to those seen in humans infected with *Onchocerca volvulus* ( Spratt & Haycock, 1988; Vuong *et al.*, 1993) *,* the causative agent of the neglected tropical disease onchocerciasis. This research prompted the hypothesis that *C. johnstoni* infection of *R. norvegicus* could provide an immunologically relevant and experimentally tractable laboratory model of onchocerciasis. Motivated by this hypothesis and progress in the development of *C. johnstoni* as a laboratory model, we have generated a draft genome assembly and annotation to understand the basic biology of the parasite. These genomic data will facilitate the investigation of hypotheses relating to host specificity, provide a resource for comparative analysis between related filarial species, and in particular, be used to characterise the genetic determinants of disease pathology and their relevance to human onchocerciasis.

## Genome sequence report

The genome was sequenced from DNA extracted from a single female parasite collected via post-mortem dissection of an Australian bush rat, *R. fuscipes*. A total of 24,374,948 300 bp paired-end reads representing ~190-fold coverage of the genome were obtained by Illumina MiSeq sequencing. Trimmed reads (n = 22,065,411) were assembled, which, after contamination and haplotype removal, resulted in an assembly with a total length of 76.9 Mbp in 2,091 scaffold sequences with a scaffold N50 of 99,003 bp and N50(n) of 232 ( Table 1). Compared to other filarial nematodes with assembled genomes, the *C. johnstoni* assembly ranked 6th of 18 based on both genome contiguity (N50) and completeness (Genome BUSCOs); we note that three assemblies with better genome contiguity and completeness statistics - *O. volvulus* ( Cotton *et al.*, 2016), *Brugia malayi* ( Foster *et al.*, 2020), and *Loa loa* (prjna246086)( Tallon *et al.*, 2014) - were all assembled using high-throughput sequencing together with one or more long molecule technologies, i.e., long-read PacBio sequencing and optical mapping, to improve contiguity whereas a further two assemblies - *L. loa* (prjna37757)( Desjardins *et al.*, 2013) and *O. flexuosa* (prjna230512) - have incorporated long-range mate-pair sequencing libraries for scaffolding. Annotation of the *C. johnstoni* genome identified 10,565 genes and 11,690 transcripts, broadly consistent with the number of reported annotation features for other filarial nematodes ( Table 1; range = 8,140-16,203 for both gene and transcript features). Similar to the genome statistics described above, the annotation of the predicted proteome is also highly resolved, with 96.5% complete BUSCOs identified ( Table 1).

**Table 1.** Genome assembly statistics of *Cercopithifilaria johnstoni* and related Clade III filarial nematodes.

Species (WBP accession ID ^1^)	Assembly length (bp)	Sequences (n)	N50 length (bp)	N50 (n)	Genome BUSCOs ^2^ (%, n=982)	Genes / transcripts (n)	Protein BUSCOs ^2^ (%, n=982)
*C. johnstoni* (current study)	76,938,880	2092	99,003	232	C:94.9 [S:94.2, D:0.7], F:3.9, M:1.2	10565, 11690	C:96.5 [S:86.5, D:10.0], F:2.3, M:1.2
*A. viteae* (prjeb1697)	77,350,906	6796	25,808	819	C:90.5 [S:88.8, D:1.7], F:7, M:2.5	10,397, 10,397	C:88.3 [S:86.5, D:1.8], F:8.8, M:2.9
*B. malayi* (prjna10729)	88,235,797	197	14,214,749	3	C:97.6 [S:96.5, D:1.1], F:1.0, M:1.4	10,928, 16,904	C:98.9 [S:71.6, D:27.3], F:0.9, M:0.2
*B. pahangi* (prjeb497)	90,545,113	14029	65,666	300	C:89.8 [S:89.2, D:0.6], F:6.6, M:3.6	14,674, 14,674	C:89.8 [S:88.7, D:1.1], F:6.7, M:3.5
*B. timori* (prjeb4663)	64,930,714	23963	4,919	3497	C:54.9 [S:54.6, D:0.3], F:20.2, M:24.9	16,203, 16,203	C:57.3 [S:56.8, D:0.5], F:20.6, M:22.1
*D. immitis* (prjeb1797)	88,309,529	16061	71,281	219	C:92.0 [S:89.8, D:2.2], F:3.8, M:4.2	12,857, 12,857	C:91.6 [S:89.2, D:2.4], F:4.3, M:4.1
*E. elaphi* (prjeb502)	82,568,297	8,078	25,590	874	C:77.6 [S:77.3, D:0.3], F:5.4, M:17.0	10,410, 10,410	C:87.5 [S:87.2, D:0.3], F:6.6, M:5.9
*L. sigmodontis* (prjeb3075)	64,813,410	3165	45,863	377	C:92.5 [S:90.6, D:1.9], F:5.1, M:2.4	10,246, 10,246	C:90.4 [S:88.2, D:2.2], F:6.7, M:2.9
*L. loa* (prjna246086)	96,405,338	2250	180,288	117	C:97.6 [S:96.3, D:1.3], F:2.0, M:0.4	12,473, 12,473	C:94.7 [S:93.4, D:1.3], F:3.5, M:1.8
*L. loa* (prjna37757)	91,373,458	5,773	174,388	130	C:96.4 [S:95.7, D:0.7], F:3.2, M:0.4	14,908, 15,445	C:96.5 [S:91.3, D:5.2], F:3.5, M:0.0
*O. flexuosa* (prjeb512)	86,175,476	45472	2,943	6666	C:48.4 [S:48.2, D:0.2], F:21.5, M:30.1	16,119, 16,119	C:67.0 [S:66.3, D:0.7], F:7.9, M:25.1
*O. flexuosa* (prjna230512)	67,740,367	1,604	540,294	22	C:72.9 [S:72.3, D:0.6], F:4.3, M:22.8	8,140, 8,235	C:67.0 [S:66.3, D:0.7], F:7.9, M:25.1
*O. ochengi* (prjeb1465)	95,513,350	24,057	12,317	1,896	C:86.3 [S:83.1, D:3.2], F:9.9, M:3.8	13,990, 13,990	C:84.7 [S:81.4, D:3.3], F:11.3, M:4.0
*O. ochengi* (prjeb1204)	91,660,559	20243	16,199	1317	C:85.5 [S:85.0, D:0.5], F:9.8, M:4.7	12,816, 12,816	C:86.2 [S:85.3, D:0.9], F:8.9, M:4.9
*O. volvulus* (prjeb513)	96,427,137	708	25,485,961	2	C:97.7 [S:97.4, D:0.3], F:1.6, M:0.7	12,109, 13,945	C:99.2 [S:98.3, D:0.9], F:0.8, M:0.0
*S. digitata* (prjna479729)	78,770,088	1,879	121,247	168	C:94.8 [S:94.3, D:0.5], F:3.6, M:1.6	10,531, 10,531	C:87.6 [S:86.6, D:1.0], F:6.2, M:6.2
*W. bancrofti* (prjeb536)	76,991,470	1350	9,917	1916	C:75.5 [S:75.1, D:0.4], F:11.6, M:12.9	13,058, 13,058	C:77.2 [S:76.7, D:0.5], F:11.1, M:11.7
*W. bancrofti* (prjna275548)	90,325,107	5105	56,670	351	C:93.5 [S:86.6, D:6.9], F:3.6, M:2.9	11,068, 11,068	C:87.4 [S:80.2, D:7.2], F:7.7, M:4.9

1 WormBase Parasite release 16 ( Howe *et al*., 2017). 2 BUSCOs: C: complete, S: complete, single copy; D: duplicated; F: fragmented; M: missing.

The immunopathology of *O. volvulus* infection is hypothesised to be driven by the recognition of immunoreactive proteins of Wolbachia ( Saint André *et al.*, 2002), a species of intracellular bacteria found in several filarial nematodes where it is thought to play a symbiotic role in host metabolism and/or reproduction ( Taylor *et al.*, 2005). The similar pathologies caused by *C. johnstoni* infection of rats and *O. volvulus* infection of humans prompted us to examine the presence of Wolbachia in our *C. johnstoni* assembly. Analysis of raw sequencing reads revealed only 0.38% of reads classified as bacterial, with less than 0.02% attributed to Rickettsiales (a group of obligate intracellular bacteria to which Wolbachia belong). Alignment of *C. johnstoni* protein-coding sequences to a diverse collection of Wolbachia reference genomes ( Lefoulon *et al.*, 2020) revealed 18 candidates; only two proteins, CJOH_00023800.t1 (blast match to YadA-like family protein) and CJOH_00083160.t1 (blast match to a prophage tail fibre N-terminal domain-containing protein / collagen-like protein) were over-represented by bacterial (but not Wolbachia specifically) relative to nematode blast hits, whereas the remaining candidates were enriched in proteins that localise to mitochondria and were present in both filaria and non-filarial nematodes. Finally, quantification of nucleotide similarity between Wolbachia and the *C. johnstoni* genome revealed that, on average, only 1.38% of the Wolbachia genome (at 65.05% nucleotide identify) was represented in sequence matches to the *C. johnstoni* scaffolds and contigs. Collectively, we conclude that Wolbachia is absent from *C. johnstoni*, and that a Wolbachia-independent mechanism drives immunopathology in *C. johnstoni* infections. 

## Methods

### Sample collection

**Figure 1.**
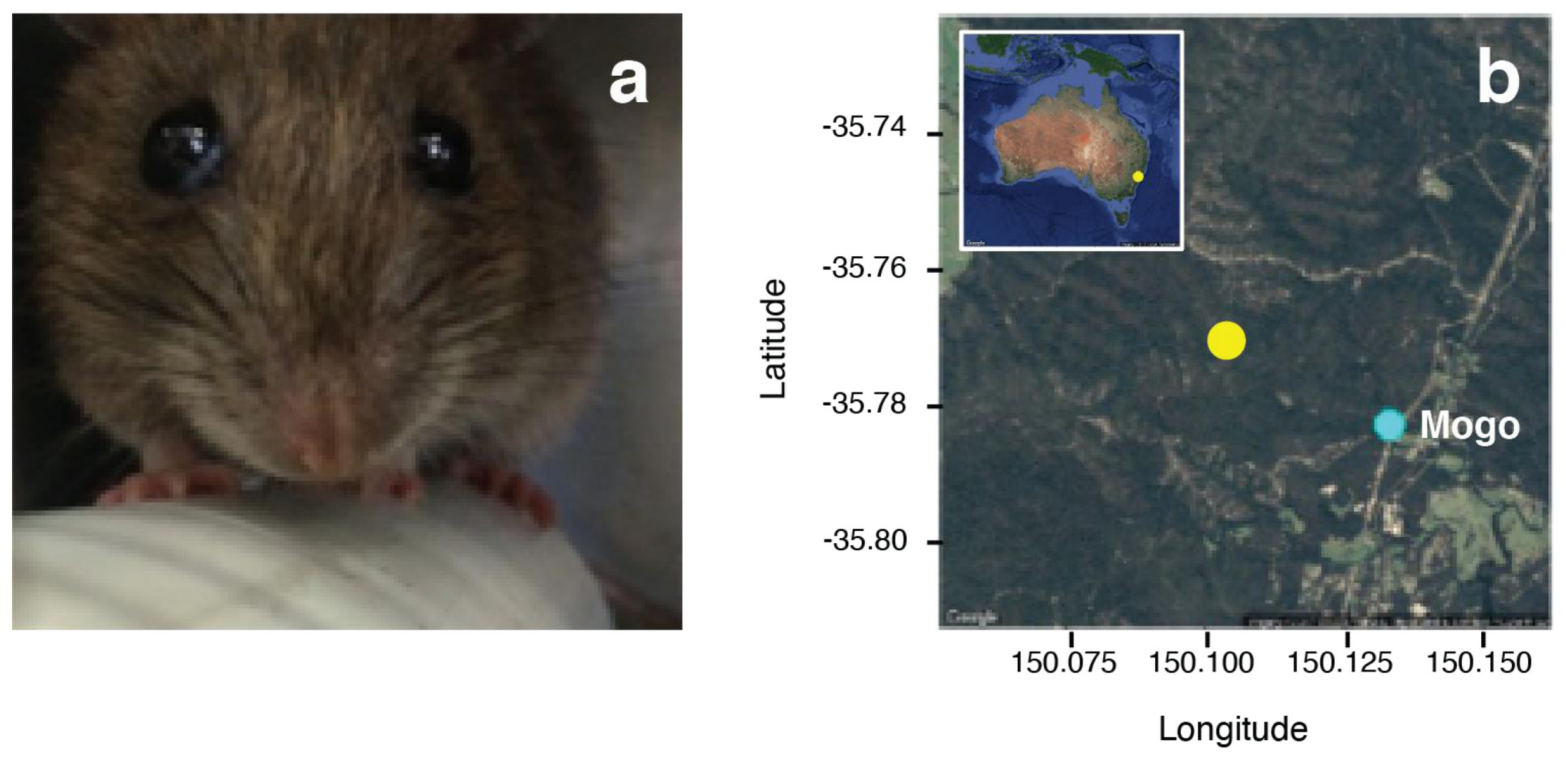
( **a**) The bush rat, *Rattus fuscipes*, is one of several host species infected by and from which *Cercopithifilaria johnstoni* used in this study were collected (photo: K. McCann). ( **b**) Sampling site (yellow point) from which bush rats were collected in the Mogo State Forest near Mogo, NSW, Australia.

As part of a larger program of fieldwork to investigate natural transmission of *C. johnstoni* in a wild, free-ranging population of Australian bush rats *Rattus fuscipes* ( Figure 1a), 8 naturally infected bush rats were transferred from the site of collection in the Mogo State Forest, N.S.W., Australia (GPS coordinates: -35.7689484, 150.1027441; Figure 1b) to the La Trobe University Animal Research Facility in Bundoora, Vic., Australia (permits: AEC 13-23, NSW – Scientific Licence 5L 101280, VIC – Scientific Permit 10007169).

All efforts were made to ameliorate any suffering of animals through providing large cages and keeping their habitat and diet as close as possible to that of the wild. The study was also closely monitored by the facility veterinarian. The rats were housed singly in large plastic tubs approximately 0.5 m × 1 m square and 1 m deep, with a hinged mesh lid. The tubs were filled with leaf litter and contained small hollow logs for refuge. Rats were fed a mix of standard rat diet supplemented with meal worms. The adult parasite that was sequenced was recovered post-mortem from a single female rat who was euthanised by CO _2_ asphyxia on advice of the facility veterinarian following a short illness of unknown origin.

### DNA extraction, library preparation, and sequencing

A single adult female worm (approximately 7 cm in length) was cut into approximately 1 cm length pieces using a sterile scalpel blade before being placed in a lysis solution (lysis buffer and proteinase K solution) for 18 h. Genomic DNA from the worm lysate was extracted using an ISOLATE II Genomic DNA Kit (Bioline, Australia) following the manufacturer’s instructions, except for the following modification: the sample was eluted from the extraction column in 50 µl of extraction buffer, which was passed back through the extraction column a second time to collect additional DNA remaining on the column before further analysis.

Genomic DNA (500 ng in 50 µl) was sheared before sequencing library preparation using a Covaris S220 Focused-ultrasonicator with the following settings optimised for generating fragments approximately 400-600 bp: Peak incidence power = 175 W; Duty factor = 5%; cycles per burst = 200; treatment time = 55 s. A DNA sequencing library was prepared from 500 ng DNA using a NEBNext Ultra Library Prep Kit for Illumina, following the manufacturer’s instructions. The resulting library was run on a 2% agarose gel, from which a gel cut was made to extract the 500-700 bp fragment fraction, which was subsequently purified using a Promega Gel and PCR clean-up kit (Promega, Australia).

The sequencing library was diluted to 15 pM and spiked with 1% PhiX control DNA (Illumina) before being sequenced on an Illumina MiSeq using Illumina V3 2x301 bp PE sequencing chemistry. In total, 24,374,948 reads (91.16% of total) passed filters and were used for further analysis.

### Genome assembly

Before assembly, raw sequencing reads were first visualised for quality and inherent bias using FastQC version 0.11.9. Reads were adapted and quality trimmed using Trimmomatic version 0.32 ( Bolger *et al.*, 2014) (CROP:150 SLIDINGWINDOW:10:20 MINLEN:100), after which 22,065,411 paired-end reads were retained for assembly. Genome size was estimated from the trimmed reads using GenomeScope 2.0 ( Ranallo-Benavidez *et al.*, 2020), which predicted a length of 63.24 Mbp.

**Figure 2.**
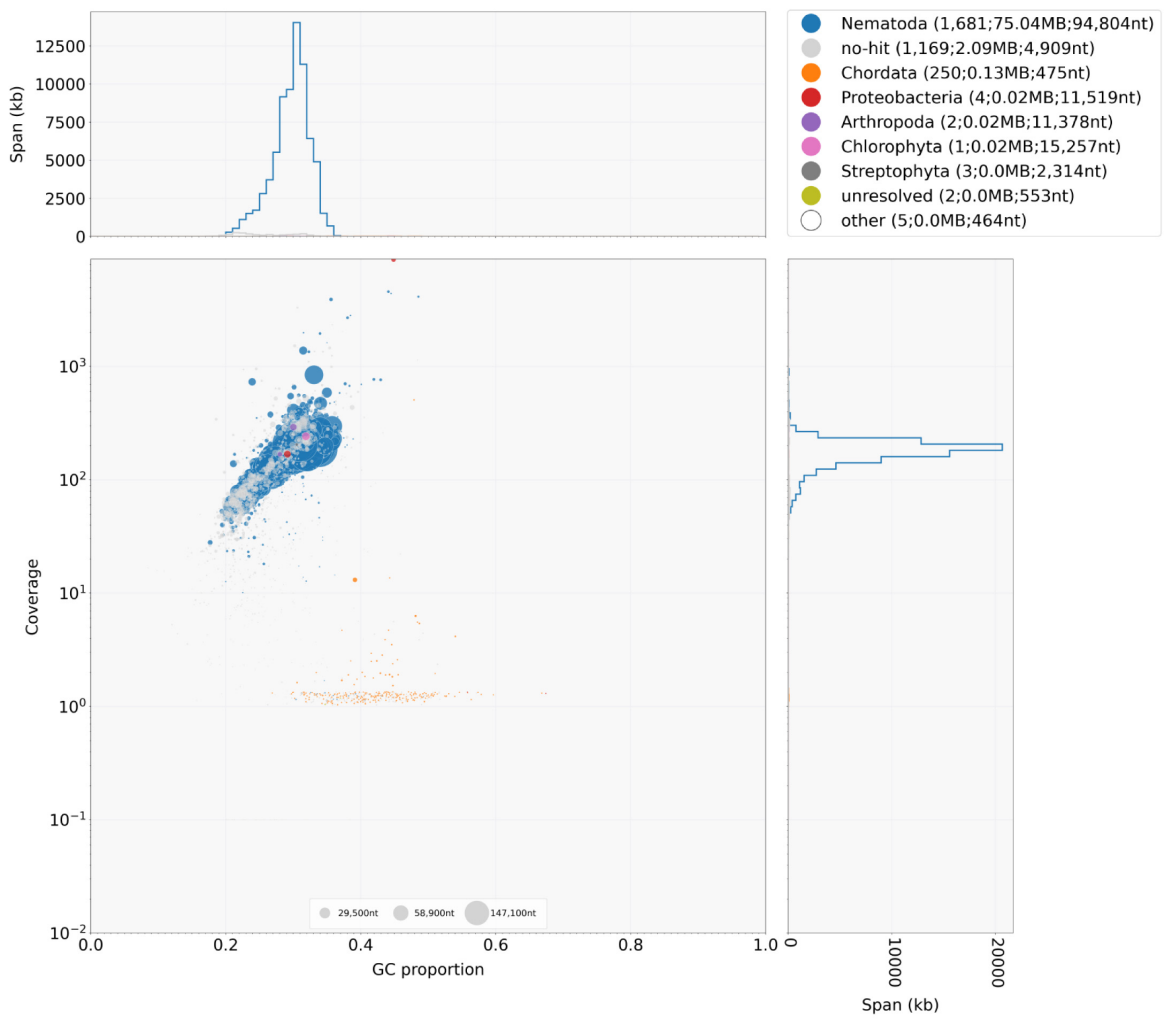
Decontamination screen using BlobTools. The plot shows variation in GC (guanine+cytosine) content (x-axis), mapped read coverage (y-axis), and blast-classification (colours, see key above) of the assembly scaffolds, from which putative contaminants are commonly identified as outliers of the distributions.

*De novo* genome assembly was performed using SPAdes version 3.10.1 ( Prjibelski *et al.*, 2020) using default parameters. The raw assembly was decontaminated, first using Redundans ( Pryszcz & Gabaldón, 2016) to remove additional haplotypes present in the assembly, followed by BlobTools ( Laetsch & Blaxter, 2017) to identify putative bacterial and host contamination present in the assembly ( Figure 2). Only scaffolds containing hits to “Nematoda” or “no-hit” (the origin of these sequences is unclear but could potentially be novel nematode sequences) and with a mapped average read depth of 10 or greater were retained. The decontaminated assembly was further scaffolded using OPERA-LG ( Gao *et al.*, 2016) to encourage unique joins that could not be previously made due to alternative haplotypes present, followed by a second-round using Redundans to fill gaps. The iterative improvements to the assembly are documented in Table 2, demonstrating improved contiguity while maintaining and recovering conserved BUSCOs.

**Table 2.** Iterative improvement of the *Cercopithifilaria johnstoni* genome assembly.

	Spades	Spades + Redundans	Spades + Redundans + Blobtools	Spades + Redundans + Blobtools + OPERA-LG	Spades + Redundans + Blobtools + OPERA-LG + gap filling (Redundans)
**Assembly statistics**					
Assembly size (bp)	79,062,707	77,312,925	77,015,453	77,032,887	76,924,992
Sequences (n)	7,152	3,117	2,568	2,263	2,091
N50 (bp)	88,758	91,012	91,596	99,003	99,003
N50 (n)	263	253	252	232	232
Average length (bp)	11,054.63	24,803.63	29,990.44	34,040.16	36,788.61
Largest scaffold (bp)	588,165	588,165	588,165	588,165	588,166
Ns (bp)	56,933	56,921	56,921	74,355	3,888
Gaps (n)	299	298	298	603	414
**Genome BUSCOs (n=982)**					
Complete	929 (94.6%)	930 (94.7%)	930 (94.7%)	930 (94.7%)	932 (94.9%)
Complete, single	922 (93.9%)	923 (94%)	923 (94%)	923 (94%)	925 (94.2%)
Complete, duplicate	7 (0.7%)	7 (0.7%)	7 (0.7%)	7 (0.7%)	7 (0.7%)
Fragmented	40 (4.1%)	39 (4.0%)	39 (4.0%)	40 (4.1%)	38 (3.9%)
Missing	13 (1.3%)	13 (1.3%)	13 (1.3%)	12 (1.2%)	12 (1.2%)

The mitochondrial genome was assembled independently of the nuclear genome. Briefly, mitochondrially-derived sequencing reads were identified by mapping all trimmed reads to mitochondrial genomes of *Onchocerca volvulus* (NC_001861.1), *Acanthocheilonema viteae* (HQ186249.1) *, Brugia malayi* (NC_004298.1) *, Dirofilaria immitis* (AJ537512.1) *, Litomosoides sigmodontis* (AP017689.1) *, Loa loa* (HQ186250.1) *, Onchocerca ochengi* (KX181290.2) *,* and *Wuchereria bancrofti* (HQ184469.1) *.* Reads that mapped were then *de novo* assembled using Velvet version 1.2.10 ( Zerbino & Birney, 2008) using default parameters, with kmer=99 identified as optimal using Velvet-optimiser version 2.2.5. Velvet was unsuccessful in producing a closed mtDNA genome, so an iterative mapping and joining approach was used to manually curate the assembly, resulting in a complete single contig of 13,716 bp. Validation of the assembly was performed by multiple sequence alignment to available filarial mtDNA genomes above using Mesquite version 3.04 ( Maddison & Maddison, 2019) and visualised in progressiveMauve (20150213) ( Darling *et al.*, 2010).

### Genome annotation

The mtDNA genome sequence was initially annotated using MITOS ( Bernt *et al.*, 2013). The *C. johnstoni* annotation was improved manually by comparing sequence alignments and GFF3 annotation files from *C. johnstoni* with the closely related filarial nematodes *L. loa*, *D. immitis*, *A. viteae*, *B. malayi*, *O. ochengi*, *O. volvulus*, *W. bancrofti*.

The nuclear genome assembly was annotated using Braker v2 ( Brůna *et al.*, 2021). As no RNA-seq data were available, we generated hints (predicted introns, start and stop codons) for Braker using the ProtHint pipeline; spliced alignments were generated by mapping proteins from OrthoDB Metazoan protein database, from which evidence (prothint_augustus.gff) was used as an input to Braker.

Annotation statistics were determined using GAG ( Geib *et al.*, 2018).

The final GFF containing both nuclear and mitochondrial genome annotations was converted to EMBL format for submission to ENA using EMBLmyGFF3 ( Norling *et al.*, 2018).

### Genome and annotation completeness

Genome and annotation completeness was estimated using BUSCO (Benchmarking Universal Single-Copy Orthologues) version 4 ( Seppey *et al.*, 2019) with lineage set to nematode_odb9 and mode set to “genome” or “protein” for the assembly or protein-coding genes, respectively, using “ *Caenorhabditis”* as a training species for gene identification. Comparative genome assembly statistics were generated using assembly-stats version 1.0.1. All genomic and proteomic data from available assemblies of related filarial nematode species were obtained from WormBase ParaSite release 16 ( Howe *et al.*, 2017).

### Wolbachia analyses

The presence of Wolbachia was assessed in three ways. First, raw sequencing reads were assessed using Kraken2 ( Wood *et al.*, 2019) against an in-house database (--db: silva_ssu_nr99_release_132). Second, all protein-coding sequences derived from the genome annotation were aligned against a diverse collection of complete Wolbachia genomes, including wMel (accession: NC_002978), wBm (NC_006833), wBp (NZ_CP050521), wCauA (CP041215), wCfeJ (NZ_CP051157.1), wCfeT (NZ_CP051156.1), wCle (NZ_AP013028), wCtub (CP046579), wDcau (CP046580), wDimm (CP046578), wFol (NZ_CP015510), wLsig (CP046577), wOo (NC_018267), wOv (NZ_HG810405), wPip (NC_010981), and wTpre (NZ_CM003641), using exonerate 2.4.0 ( Slater & Birney, 2005), from which hits were queried using BLASTP. Finally, the relative proportion of Wolbachia genome sequence matches to the *C. johnstoni* assembly was quantified using PROmer version 3.07 ( Kurtz *et al.*, 2004).

The analysis code used in this study is available from GitHub and is archived with Zenodo ( Doyle & McKann, 2021).

## Data availability

### Genomic resources

European Nucleotide Archive: Raw sequence data, genome and annotation are deposited in the ENA. Accession number PRJEB47283; https://identifiers.org/ena.embl:PRJEB47283.

The assembly will also be made available at WormBase ParaSite ( https://parasite.wormbase.org/), the primary repository for helminth genomes and annotations.

### Analysis code

Analysis code is available from: https://github.com/stephenrdoyle/cercopithifilaria_johnstoni.

Archive analysis code at time of publication: https://doi.org/10.5281/zenodo.5545956 ( Doyle & McKann, 2021).

License: BSD 3-Clause “New” or “Revised” License
