# Supplementary material for: The genome sequence of the Australian filarial nematode, Cercopithifilaria johnstoni
Source: Wellcome Open Res. 2021 Dec 3;6:259. Originally published 2021 Oct 12. [Version 2] doi: 10.12688/wellcomeopenres.17258.2 (PMC8564745; doi:10.12688/wellcomeopenres.17258.2)
Supplement: Peer review history

# Peer review: Reviewer response for version 2

**DOI:** 10.21956/wellcomeopenres.19331.r47422

**Published:** 2021-12-06

**Authors:** James Wasmuth

**Affiliations:** 1 Department of Ecosystem and Public Health, Faculty of Veterinary Medicine, University of Calgary, Calgary, Canada; 2 Host-Parasite Interactions (HPI) Research Training Network, University of Calgary, Calgary, Canada

I thank the authors for their careful consideration of my comments. The additional information they provide in the publication easily satisfies my earlier concerns.

Are sufficient details of methods and materials provided to allow replication by others?

Yes

Is the rationale for creating the dataset(s) clearly described?

Yes

Are the datasets clearly presented in a useable and accessible format?

Yes

Are the protocols appropriate and is the work technically sound?

Yes

Reviewer Expertise:

Genome informatics, nematode evolution

I confirm that I have read this submission and believe that I have an appropriate level of expertise to confirm that it is of an acceptable scientific standard.

# Peer review: Reviewer response for version 2

**DOI:** 10.21956/wellcomeopenres.19331.r47424

**Published:** 2021-12-06

**Authors:** Neil Young

**Affiliations:** 1 Department of Veterinary Biosciences, Melbourne Veterinary School, The University of Melbourne, Melbourne, Vic, Australia

Thanks for the comprehensive response to the comments. I believe the version 2 of the manuscript addresses the aims/objectives clearly. It also provides more detail on the position of this nematode to guide the use of this resource for future comparative genomic and genetic studies.

Are sufficient details of methods and materials provided to allow replication by others?

Yes

Is the rationale for creating the dataset(s) clearly described?

Yes

Are the datasets clearly presented in a useable and accessible format?

Yes

Are the protocols appropriate and is the work technically sound?

Yes

Reviewer Expertise:

Parasite genomics and genetics.

I confirm that I have read this submission and believe that I have an appropriate level of expertise to confirm that it is of an acceptable scientific standard.

# Peer review: Reviewer response for version 1

**DOI:** 10.21956/wellcomeopenres.19074.r46395

**Published:** 2021-11-02

**Authors:** Neil Young

**Affiliations:** 1 Department of Veterinary Biosciences, Melbourne Veterinary School, The University of Melbourne, Melbourne, Vic, Australia

The article by McKann *et al.* reports an annotated draft genome of *Cercopithifilaria johnstoni, *a filarial nematode parasitising Australian mammals. Despite the fragmented nature of the assembly, the genome is of significant value to the research community because of its taxonomic position, its adaptive evolution to parasitise marsupials and its potential use as a laboratory model system for onchocerchiasis. Combined fundamental and applied applications make it a valuable nematode genomic resource.

 I was surprised to see genome completeness scores over 94% with an assembly using only short-read sequence data. Could this relate to short introns with less repetitive elements?

 As this parasite is a useful model for onchocerciasis, it would be good to show the completeness of gene models specific to parasite-host interactions in *Onchocerca *and related species.

 The mt genome was also assembled and annotated but there was no description of the mt genome herein. A mt phylogenomic tree might provide the reader with more context of the taxonomic position of this parasite.

 Did the lack of RNAseq data affect gene model predictions? If not, then the findings herein would be strong support for relying only on amino acid sequence homology for training ab initio gene predictors. It would simplify efforts to complete the genome annotations for some taxa.

Are sufficient details of methods and materials provided to allow replication by others?

Yes

Is the rationale for creating the dataset(s) clearly described?

Yes

Are the datasets clearly presented in a useable and accessible format?

Yes

Are the protocols appropriate and is the work technically sound?

Yes

Reviewer Expertise:

Parasite genomics and genetics.

I confirm that I have read this submission and believe that I have an appropriate level of expertise to confirm that it is of an acceptable scientific standard, however I have significant reservations, as outlined above.

# Response

**Published:** 2021-12-01

**Authors:** Stephen Doyle

**Reviewer 3 – Neil Young**

 The article by McKann *et al.* reports an annotated draft genome of Cercopithifilaria johnstoni, a filarial nematode parasitising Australian mammals. Despite the fragmented nature of the assembly, the genome is of significant value to the research community because of its taxonomic position, its adaptive evolution to parasitise marsupials and its potential use as a laboratory model system for onchocerchiasis. Combined fundamental and applied applications make it a valuable nematode genomic resource.

 I was surprised to see genome completeness scores over 94% with an assembly using only short-read sequence data. Could this relate to short introns with less repetitive elements?

***Response: We were also pleasantly surprised by the relatively high genome completeness statistics for an Illumina-only assembly. We attribute it in part to the low diversity of sequencing a freshly-collected individual parasite at high coverage with relatively long Illumina reads (3x300 bp). The genome at 76 Mb is on the smaller side compared to other nematodes, and while its overall repetitive content was not noticeably different from other filarial nematodes, it must compact its genome content into a smaller space suggesting it is “less complex” to some degree.***

******

***Regarding the intron lengths - we do find that C. johnstoni introns are shorter than O. volvulus introns (See table below on intron length stats). To what extent this is due to assembly contiguity (i.e., a technical effect - the O. volvulus genome is assembled into chromosomal-scale scaffolds and so provides a more robust framework for longer gene models), or true biological differences, is difficult to determine without having a more contiguous C. johnstoni assembly.***

 | Species | count | mean_length | stdev   | median_length | q1    | q3  |

 |------------|----------|------------------|-----------|----------------------|-------|------|

 |  OV       | 93854 | 435.9            | 2311.6 | 257                    | 155 | 409 |

 |  CJ        | 97658 | 299.0            | 298.73 | 229                   | 143 | 350 |

 As this parasite is a useful model for onchocerciasis, it would be good to show the completeness of gene models specific to parasite-host interactions in Onchocerca and related species.

***Response: We provide an estimate of the overall geneset completeness, as indicated by the BUSCO scores for the genome and proteome in Table 1. Arguably, these data show that the genome and proteome are highly representative based on conserved orthologs “expected” to be present, and relative to closely related species. We agree that to further establish C. johnstoni as a model for onchocerciasis, a better understanding of the genes involved in host-parasite interactions is needed; these data are in fact the focus of a separate follow up publication. As a Wellcome Open Research Data Note aims to focus specifically on the data themselves and “not… analyses or conclusions”, we initially (and now again, subsequently after peer review) decided against presenting these downstream analyses of the genome resources.***

 The mt genome was also assembled and annotated but there was no description of the mt genome herein. A mt phylogenomic tree might provide the reader with more context of the taxonomic position of this parasite.

***Response: The reviewer is correct – we did not specifically describe the mitochondrial genome. However, we agree that a phylogeny using the mitochondrial genome would illustrate where C. johnstoni is placed relative to other filarial species.***

******

***To address this comment, we now include this phylogeny in Figure 3.***

 Did the lack of RNAseq data affect gene model predictions? If not, then the findings herein would be strong support for relying only on amino acid sequence homology for training ab initio gene predictors. It would simplify efforts to complete the genome annotations for some taxa.

***Response: This is a difficult question to respond to specifically, given we didn’t generate RNA-seq data for C. johnstoni. However, the BUSCO predictions were respectable given we did use amino acid homology from a broad range of metazoan species.***

******

***To explore this idea as a purely academic exercise, we reannotated the genomes of a closely related but less-well annotated species (Acanthocheilonema viteae) and a species with a high-quality genome / good annotation (Onchocerca volvulus). The predicted proteins inferred from these new annotations were assessed using BUSCO. We note that both A. viteae and O. volvulus are not (yet) included in the OrthoDB database from which the metazoan proteins used to generate the hints were derived; thus, the de novo annotations described below are not biased by pre-existing species-specific protein models.***

******

***A. viteae***

***-  Genes:***

***o   original: 10,397***

***o   de novo: 12,056***

***-  BUSCOS:***

***o   original: C: 88.3 [S:86.5, D:1.8], F:8.8, M:2.9***

***o   de novo: C: 92.4 [S:82.6, D:9.8], F:5.6, M:2.0***

***-  Conclusion: New models are more complete, less fragmented, fewer missing, however, there is a higher duplication rate, which may relate to new alternative transcripts present.***

******

***O. volvulus***

***-  Genes***

***o   original: 12,109***

***o   de novo: 12473***

***-  BUSCOs:***

***o   original: C:99.2 [S:98.3, D:0.9], F:0.8, M:0.0***

***o   de novo: C:98.8 [S:88.9, D:9.9], F:1.0, M:0.2***

***-  Conclusion: Minor differences overall - slightly fewer complete models, slightly more fragmented, and slightly more missing. Higher duplication rates. However, O. volvulus is outstanding already, and the de novo annotation is still very good overall.***

******

***These results suggest, based on a relatively simple metric of the proportion of conserved orthologous genes, that using a large collection of diverse metazoan proteins as hints for Braker2 can improve existing annotations and does a respectable job when compared with a well-curated genome annotation. Therefore, it is likely that this represents a valid approach for annotation of genomes from species where collecting additional species-species evidence, ie, RNA-seq, is difficult. This needs further testing, which is outside the scope of this work.***

******

***This validation exercise of the approach used to annotate the C. johnstoni genome as we describe provides further support for the high BUSCO scores we report and completeness of the C. johnstoni genome and annotation.***

# Peer review: Reviewer response for version 1

**DOI:** 10.21956/wellcomeopenres.19074.r46393

**Published:** 2021-10-27

**Authors:** Jane Hodgkinson

**Affiliations:** 1 Veterinary Parasitology, Institute of Infection, Veterinary and Ecological Sciences, University of Liverpool, Liverpool, UK

The authors present a genome for the nematode *Cercopithifilaria johnstoni, *a parasite of considerable interest in its own right and as a comparator for other filarial nematodes.

 In my opinion all the methodologies are appropriate and every attempt has been made to produce a genome assembly of the best quality with the available sequence data. Table 1 clearly identifies that the quality of the assembly of *Cercopithifilaria johnstoni *as presented, is comparable with the quality of published genomes for other filaria; indeed it is towards the top end (6/18) in terms of completeness and contiguity.

 I have no reservations in recommended this manuscript for indexing in its current form.

Are sufficient details of methods and materials provided to allow replication by others?

Yes

Is the rationale for creating the dataset(s) clearly described?

Yes

Are the datasets clearly presented in a useable and accessible format?

Yes

Are the protocols appropriate and is the work technically sound?

Yes

Reviewer Expertise:

Molecular helminthology, anthelmintic resistance

I confirm that I have read this submission and believe that I have an appropriate level of expertise to confirm that it is of an acceptable scientific standard.

# Response

**Published:** 2021-12-01

**Authors:** Stephen Doyle

***Response: We are grateful for the positive appraisal of our work.***

# Peer review: Reviewer response for version 1

**DOI:** 10.21956/wellcomeopenres.19074.r46394

**Published:** 2021-10-26

**Authors:** James Wasmuth

**Affiliations:** 1 Department of Ecosystem and Public Health, Faculty of Veterinary Medicine, University of Calgary, Calgary, Canada; 2 Host-Parasite Interactions (HPI) Research Training Network, University of Calgary, Calgary, Canada

The authors have done an excellent job in describing the sequencing, assembly and annotation of the genome of a parasitic nematode, whose broad host range recommends it for understanding how host-parasite interactions evolve. While the sequencing is only short-read (300 bp), the depth of sequencing and careful assembly gives us confidence in the gene models. The level of detail in the methods should be considered the new standard of reporting. I enjoyed the data in table 2, which demonstrates the value of careful assembly. I have three requests in any future version:

 1. It would be helpful to know the phylogenetic placement of *C. johnstoni* in the filarial nematodes from this paper. Perhaps Table 1 could include the phylogenetic relationships.

 2a. In the search for *Wolbachia* in *C. johnstoni* the authors found that 0.02% of reads mapped to Rickettsiales. What is the % of reads from the *O. volvulus* sequencing project that maps to Rickettsiales? This comparison is necessary as this report will be cited as evidence of *Wolbachia* loss.

 2b. For the other *Wolbachia* searches, it is unclear to me if the authors used the assembled contigs before or after blobtools decontamination. If after, it is not surprising that there is so little evidence of matches.

Are sufficient details of methods and materials provided to allow replication by others?

Yes

Is the rationale for creating the dataset(s) clearly described?

Yes

Are the datasets clearly presented in a useable and accessible format?

Yes

Are the protocols appropriate and is the work technically sound?

Yes

Reviewer Expertise:

Genome informatics, nematode evolution

I confirm that I have read this submission and believe that I have an appropriate level of expertise to confirm that it is of an acceptable scientific standard, however I have significant reservations, as outlined above.

# Response

**Published:** 2021-12-01

**Authors:** Stephen Doyle

**Reviewer 1 – James Wasmuth**

 The authors have done an excellent job in describing the sequencing, assembly and annotation of the genome of a parasitic nematode, whose broad host range recommends it for understanding how host-parasite interactions evolve. While the sequencing is only short-read (300 bp), the depth of sequencing and careful assembly gives us confidence in the gene models. The level of detail in the methods should be considered the new standard of reporting. I enjoyed the data in table 2, which demonstrates the value of careful assembly.

 I have three requests in any future version:

 1. It would be helpful to know the phylogenetic placement of C. johnstoni in the filarial nematodes from this paper. Perhaps Table 1 could include the phylogenetic relationships.

***Response: We agree that this is would be useful to show.***

******

***We have now included a phylogeny of filarial species based on whole mitochondrial genome alignment as Figure 3.***

******

 2a. In the search for Wolbachia in C. johnstoni the authors found that 0.02% of reads mapped to Rickettsiales. What is the % of reads from the O. volvulus sequencing project that maps to Rickettsiales? This comparison is necessary as this report will be cited as evidence of Wolbachia loss.

***Response: This is a really good question and one that we had not originally asked.***

******

***To address this comment, we determined the proportion of reads classified as Wolbachia from 32 O. volvulus whole-genome sequencing datasets described in Choi et al. 2016 ( https://doi.org/10.1038/nmicrobiol.2016.207). We also improved the sensitivity of analysis using a new custom kraken database with all known filarial Wolbachia genomes added to it, including the Wolbachia genome from O. volvulus. This was important, as an initial analysis of a single O. volvulus read set performed poorly with the original kraken database used.***

******

***The new analysis revealed only a single C. johnstoni sequencing read classified as Wolbachia (rather than Rickettsiales as we reported originally), whereas, on average, 1.98% of O. volvulus reads classified as Wolbachia (range: 0.08-13.26%, average library size = 34 million reads). Considering the O. volvulus Wolbachia genome is ~1 Mb and the nuclear genome ~100 Mb, it suggests there are ~ 2 Wolbachia genomes for every nuclear genome, which is within the range we have observed previously estimated from mapped reads to the nuclear and Wolbachia genomes (see https://doi.org/10.1186/s13071-017-2126-4).***

******

***These new results are now included in the manuscript.***

 2b. For the other Wolbachia searches, it is unclear to me if the authors used the assembled contigs before or after blobtools decontamination. If after, it is not surprising that there is so little evidence of matches.

***Response: Well observed. We originally analysed matches between Wolbachia and the assembled genomes pre- and post-decontamination, but in the end, only reported the analysis of the final genome assembly.***

******

***To address this comment, we now report the analysis performed on the Spades assembly prior to blobtools processing; encouragingly, we find only 1.4% of Wolbachia matches in the Spades-only assembly, which is consistent with the 1.38% we originally reported using the decontaminated genome.***

******

***Collectively, these results strengthen our argument that C. johnstoni does not harbour Wolbachia.***
